# Lysosomal-Associated Protein Transmembrane 5 Functions as a Novel Negative Regulator of Pathological Cardiac Hypertrophy

**DOI:** 10.3389/fcvm.2021.740526

**Published:** 2021-10-06

**Authors:** Lu Gao, Sen Guo, Rui Long, Lili Xiao, Rui Yao, Xiaolin Zheng, Yanzhou Zhang, Xiaofang Wang

**Affiliations:** ^1^Department of Cardiology, The First Affiliated Hospital of Zhengzhou University, Zhengzhou, China; ^2^Department of Geriatrics, The First Affiliated Hospital of Zhengzhou University, Zhengzhou, China

**Keywords:** LAPTM5, cardiac hypertrophy, signal transduction, Rac1, MEK-ERK pathway

## Abstract

Lysosomal-associated protein transmembrane 5 (LAPTM5) is mainly expressed in immune cells and has been reported to regulate inflammation, apoptosis and autophagy. Although LAPTM5 is expressed in the heart, whether LAPTM5 plays a role in regulating cardiac function remains unknown. Here, we show that the expression of LAPTM5 is dramatically decreased in murine hypertrophic hearts and isolated hypertrophic cardiomyocytes. In this study, we investigated the role of LAPTM5 in pathological cardiac hypertrophy and its possible mechanism. Our results show that LAPTM5 gene deletion significantly exacerbates cardiac remodeling, which can be demonstrated by reduced myocardial hypertrophy, fibrosis, ventricular dilation and preserved ejection function, whereas the opposite phenotype was observed in LAPTM5 overexpression mice. In line with the *in vivo* results, knockdown of LAPTM5 exaggerated angiotensin II-induced cardiomyocyte hypertrophy in neonatal rat ventricular myocytes, whereas overexpression of LAPTM5 protected against angiotensin II-induced cardiomyocyte hypertrophy *in vitro*. Mechanistically, LAPTM5 directly bound to Rac1 and further inhibited MEK-ERK1/2 signaling, which ultimately regulated the development of cardiac hypertrophy. In addition, the antihypertrophic effect of LAPTM5 was largely blocked by constitutively active mutant Rac1 (G12V). In conclusion, our results suggest that LAPTM5 is involved in pathological cardiac hypertrophy and that targeting LAPTM5 has great therapeutic potential in the treatment of pathological cardiac hypertrophy.

## Introduction

Pathological cardiac hypertrophy is a common pathological process of heart failure. Its main feature is the enlargement of cardiomyocytes, leading to cardiac systolic dysfunction (1–3). Although the initial pathological hypertrophic response is a compensatory change (cardiac output can be maintained), the beneficial effects are contravened by sustained pathological hypertrophy, which eventually results in malignant arrhythmia, heart failure, and sudden death. Although numerous studies have implicated multiple regulatory networks in the progression of cardiac hypertrophy, the underlying mechanisms of cardiac hypertrophy and the resultant heart failure remain unclear (4–6). Therefore, an in-depth understanding of the pathogenesis of cardiac hypertrophy is very important to determine new therapeutic targets.

Lysosome-associated protein transmembrane 5 (LAPTM5) is mainly expressed in lymphoid and bone marrow-derived cells and can interact with Nedd4, a member of the E3 ubiquitin ligase family. This appears to be the most important and the best studied function of LAPTM5 (7, 8). In addition, the decreased expression of LAPTM5 can inhibit the ectopic overexpression of LAPTM5 and induce apoptosis (9, 10). Recent studies have indicated that LAPTM5 positively regulates proinflammatory signaling pathways and proinflammatory cytokine production in macrophages (11). In addition, recent studies have found that LAPTM5 is involved in the regulation of several signaling pathways, including nuclear factors-κB (NF-κB), transforming growth factor β (TGFβ)-Smads, phosphoinositide 3-kinase (PI3K)-AKT, and MAPKs, which are associated with cardiovascular diseases (11–13). These studies suggest that LAPTM5 may play a role in cardiovascular disease. Moreover, studies have shown that LAPTM5 is expressed in the heart, but its function in the heart is still unclear. Thus, we are interested in determining whether LAPTM5 can function as a regulator in cardiovascular diseases, especially the development of cardiac hypertrophy and heart failure.

In this study, we first demonstrated that the levels of LAPTM5 were downregulated in cardiomyocytes and mouse hearts subjected to hypertrophic stress. In addition, deletion of the LAPTM5 gene aggravated pressure load-induced myocardial hypertrophy and heart failure in mice. However, overexpression of LAPTM5 protein in the heart can reduce myocardial hypertrophy and cardiac dysfunction induced by chronic pressure overload. At the molecular level, we identified that the beneficial effect of LAPTM5 on cardiac hypertrophy was largely dependent on the regulation of the Rac1-MEK-ERK1/2 signaling pathway. Furthermore, the antihypertrophic effect of LAPTM5 was largely blocked by constitutively active mutant Rac1 (G12V). Collectively, our results delineated that LAPTM5 ameliorates the development of pathological cardiac hypertrophy by interacting with Rac1 and then blocking the activation of the Rac1-dependent signaling cascade.

## Materials and Methods

### Experimental Animals

The Animal Care and Use Committee of the First Affiliated Hospital of Zhengzhou University approved all animal protocols. The animal experiments were conducted in accordance with the National Institutes of Health Guidelines for the Care and Use of Laboratory Animals (NIH Publication No. 80–23, revised in 1996).

### LAPTM5 Knockout Mouse Construction

Single guide RNA (sgRNA) targeting CCAGGGCTATGGTGGCGACT in the first exon of LAPTM5 was designed and cloned in the BsaI restriction site of the pUC57-T7-sgRNA (Addgene, 51132). Cas9 mRNA and guide RNA (gRNA) were generated by *in vitro* transcription following the instructions (Thermo Fisher Scientific, USA) and co-microinjected into the cytoplasm of fertilized eggs collected from C57BL/6N mice at the one-cell stage. The injected embryos were then implanted into the oviducts of pseudopregnant foster mothers. Two-week-old newborn mice were subjected to DNA extraction from the ear tissue and genotyped through sequencing PCR-amplified products with the following primers: LAPTM5-check F1: 5′- GGGCCCAAGACTCCTTACTC-3′, LAPTM5-check R1: 5′- CCCAGACTCCCCAATACTCA -3′.

### AAV9-Viral-Based Gene Delivery in Mice

AAV9-LAPTM5 (also called Ad-LAPTM5) and AAV9-Vector (as a negative control, also called Ad-NC) were constructed as described above. In brief, the LAPTM5 cassette consists of a CMV promoter followed by the human LAPTM5 sequence, and LAPTM5 was cloned into a pAAV-Vector. Then, the recombinant plasmid was identified by RT–PCR 24 h later. Next, the recombinant plasmids together with Helper and pAAV-RC were cotransfected into AAV-293 cells. After 3 days, viral stocks were obtained by CsCL2 gradient centrifugation. Finally, RT–PCR was used to detect the titration of aav9-LAPTM5 and AAV9-Vector. Two weeks before AB, mice were injected *via* the tail vein with virus containing 7.5 × 10^11^ VG of the AAV9-LAPTM5 or AAV9-Vector.

### Mouse Aortic Banding Surgery and Echocardiographic Measurements

AB surgery was performed as we previously described (14, 15). The mice were depilated and fasted for 12 h before the operation, and the mice were anesthetized by intraperitoneal injection of pentobarbital. In the AB groups, the aortic arch was separated and ligated. In the sham groups, the aortic arch was isolated without ligation. The appropriate ligation was evaluated by Doppler ultrasound.

A high-resolution small animal ultrasound system was used to evaluate the structure and function of the mouse heart as we previously described (16). The left ventricular end systolic diameter (LVESD) and left ventricular end diastolic diameter (LVEDd) were measured by M-mode. The contraction function is reflected by calculating the left ventricular fraction shortening (LVFS). All parameters were measured by M-mode for at least 5 cardiac cycles and then averaged.

### Histological Analysis

The mice were euthanized at 4 weeks after AB or sham surgery to assess hypertrophic growth and cardiac fibrosis. Paraffin-embedded heart sections were stained with hematoxylin and eosin (H&E) for histopathology or with picrosirius red (PSR) to clarify collagen deposition. A fluorescence microscope was used to acquire images, and a digital image analysis system (Image-Pro Plus 6.0) was used to measure individual cell sizes. The LV collagen volume fraction was calculated from the PSR-stained sections as the area stained by PSR divided by the total area by Image-Pro Plus 6.0.

### Lentivirus Construction and Transfection, Neonatal Rat Cardiomyocyte Culture, and Immunofluorescent Staining

Lentiviruses used for overexpressing LAPTM5 (Lenti-LAPTM5) or LAPTM5 knockdown (LentishLAPTM5) were constructed. Briefly, full-length LAPTM5 coding cDNA was inserted into the lentiviral vector construct downstream of a cytomegalovirus (CMV) promoter to overexpress LAPTM5. Three pairs of oligo sequences targeting rat LAPTM5 mRNA containing different candidate short hairpin RNA sequences were synthesized, denatured and separately ligated into the pLKO1 lentiviral vector downstream of the U6 promoter to produce Lenti-LAPTM5. The lentivirus vector construct, packaging construct and envelope plasmid were cotransfected into 293T cells to prepare lentivirus particles, and the virus was stored at −80°C until use.

In particular, three lines of Lenti-LAPTM5 were screened for their efficiency in LAPTM5 knockdown, and the lentiviral line that produced the highest reduction in LAPTM5 protein levels was utilized. Lentivirus expressing GFP protein was used as a control for Lenti-LAPTM5, and a scramble Lenti-shRNA was used as a control for Lenti-shLAPTM5.

Primary neonatal rat cardiomyocyte culture, lentivirus transfection and Ang II stimulation were performed as we previously described (16). In brief, NRCMs were isolated from the hearts of 1- to 2-day-old Sprague–Dawley rat pups and cultured in gelatin-coated six-well plates. Lentivirus was added at a multiplicity of infection (MOI) of 10 to transfect NRCMs in serum-free medium for 6 h. Then, these cells were maintained for 72 h, synchronized with serum-free medium and stressed with Ang II (1 μmol/L) for the indicated hours. Immunofluorescence staining of α-actinin (Sigma–Aldrich, A7811) in cultured NRCMs was performed using established protocols.

### Real-Time PCR

Total RNA was isolated and purified as described in our previous study (16). The SuperScript first strand complementary DNA system (Invitrogen) was used for reverse transcription. PCR amplifications were quantified using SYBR Green PCR Master Mix (Applied Biosystems) and normalized to GAPDH gene expression. Primer sequences used for RT–PCR listed in Table 1.

**Table 1:** Primer sequences used for RT–PCR.

**mRNA**	**Forward**	**Reverse**
ANP^a^	ACCTGCTAGACCACCTGGAG	CCTTGGCTGTTATCTTCGGTACCGG
BNP^a^	GAGGTCACTCCTATCCTCTGG	GCCATTTCCTCCGACTTTTCTC
β-MHC^a^	CCGAGTCCCAGGTCAACAA	CTTCACGGGCACCCTTGGA
Col1agenI^a^	AGGCTTCAGTGGTTTGGATG	CACCAACAGCACCATCGTTA
Col1agenIII^a^	AAGGCTGCAAGATGGATGCT	GTGCTTACGTGGGACAGTCA
CTGF^a^	AGGGCCTCTTCTGCGATTTC	CTTTGGAAGGACTCACCGCT
GAPDH^a^	ACTCCACTCACGGCAAATTC	TCTCCATGGTGGTGAAGACA
ANP^b^	AAAGCAAACTGAGGGCTCTGCTCG	TTCGGTACCGGAAGCTGTTGCA
β-MHC^b^	TCTGGACAGCTCCCCATTCT	CAAGGCTAACCTGGAGAAGATG
GAPDH ^b^	GACATGCCGCCTGGAGAAAC	AGCCCAGGATGCCCTTTAGT

*Sequences are listed 5′−3′.*

a *The PCR used the primers in mice.*

b *PCR using primers in neonatal rat cardiomyocytes.*

### Western Blot Assay

Total proteins were extracted as described in our previous study (16), and the protein concentration was determined with a BCA protein assay kit. Protein extracts (50 μg) were separated by SDS–PAGE and transferred to nitrocellulose membranes and probed with various primary antibodies, including β-MHC, LAPTM5 (from Abcam, 1:1,000 dilution), ANP (from Santa Cruz, 1:200 dilution), GAPDH, P-MEK1/2, T-MEK1/2, P-ERK1/2, ERK1/2, P-JNK1/2, JNK1/2, P-P38, and P38 (from Cell Signaling Technology, 1:1000 dilution). After incubation with a secondary IRDye^®^ 800CW-conjugated antibody, Image Lab 5.2.1 software was used for quantification. GAPDH was used as a reference protein.

### Co-immunoprecipitation Assay

For the Co-IP assay, the treated cells were collected, and IP lysate was added to lyse the cells. The cell lysate was added with the corresponding antibody and A/G-Agarose beads for incubation. After the immunoprecipitation reaction, the immune complex was collected and eluted from agarose beads to free antigen, antibody and beads. Finally, Western blotting was performed to determine the binding protein.

### GST Pull-Down Assay

The GST-fused full-length and truncated forms of LAPTM5 and Rac1 were expressed in Rosetta (DE3) *Escherichia coli* and purified after being immobilized on glutathione-sepharose 4B beads (GE Healthcare). Then, protein-bound beads were incubated with Flag-Rac1- or Flag-LAPTM5-expressing HEK293T cell lysates in IP buffer (20 mM Tris-HCl, pH 8.0, 150 mM NaCl, 1 mM EDTA and 0.5% NP-40 supplemented with protease inhibitor cocktail) for 4 h at 4°C. The beads were then washed four times with IP lysis buffer in the absence of protease inhibitor cocktail. Finally, the bound proteins on the beads were eluted, resolved by SDS–PAGE and analyzed by Western blotting.

### Confocal Microscopy

HEK293T cells were cultured on gelatin-coated coverslips and cotransfected with pEGFP-LAPTM5 and pmCherry-Rac1 for 48 h. Then, the cells were fixed with paraformaldehyde, permeabilized in PBS with Triton X-100, and stained with DAPI. The slides were fixed with fixed solution and finally observed and photographed with a confocal laser scanning microscope.

### Statistical Analysis

Data are shown as means ± SD. Differences among groups were assessed by ANOVA followed by Tukey's *post-hoc* test. Comparisons between two groups were performed by Student's *t*-test. A value of *P* < 0.05 was considered to be a statistically significant difference.

## Results

### LAPTM5 Expression Is Upregulated by Hypertrophic Stimuli

**Figure 1:**
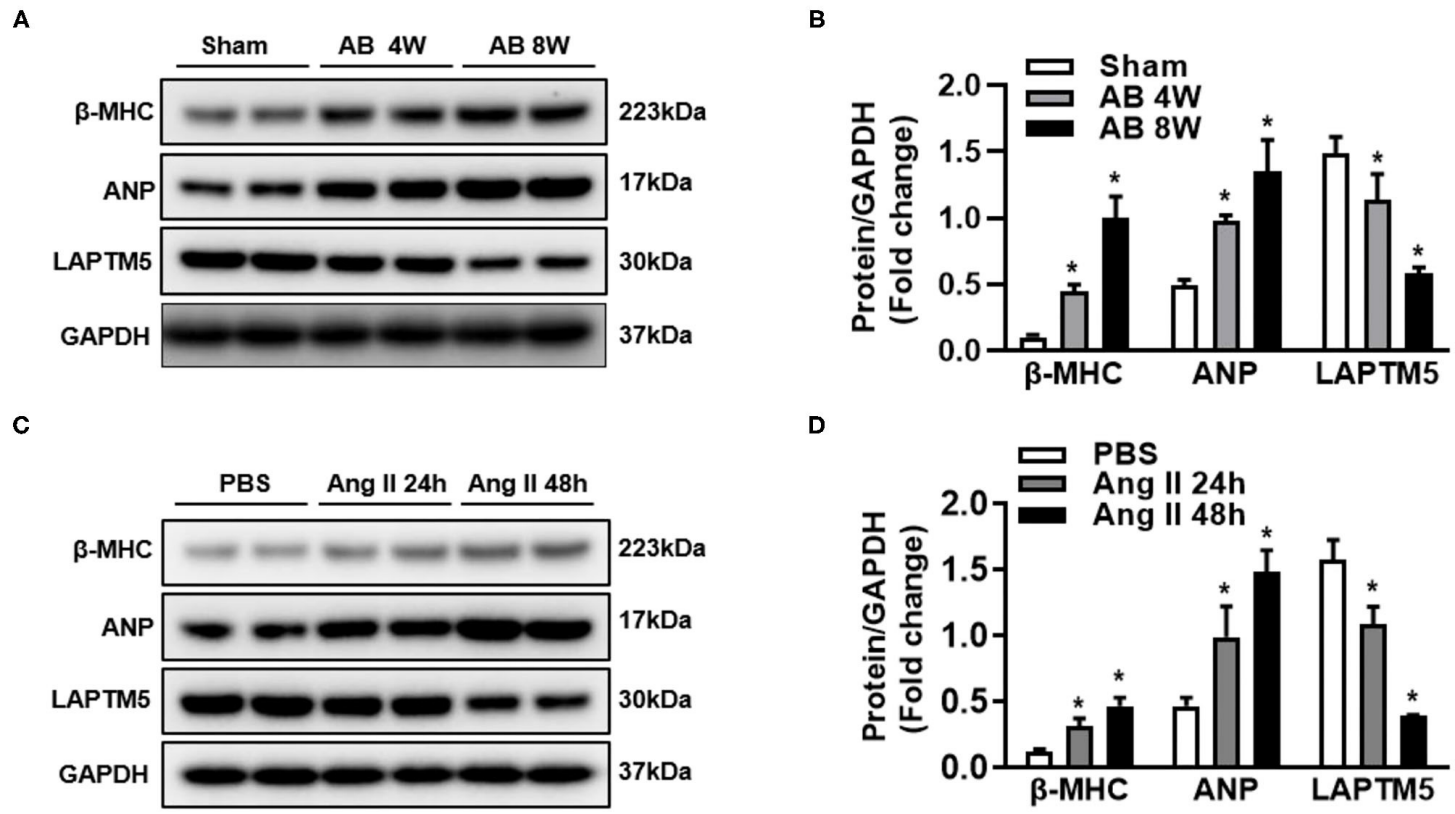
Expression of LAPTM5 is downregulated in experimental hypertrophic models. **(A,B)** Western blot analysis of β-myosin heavy chain (β-MHC), atrial natriuretic peptide (ANP) and LAPTM5 levels in hearts from mice subjected to sham or aortic banding (AB) surgery (^*^P < 0.05 vs. sham). **(C,D)** β-MHC, ANP and LAPTM5 levels in neonatal rat cardiomyocytes treated with phosphate buffered saline (PBS) or angiotensin II (Ang II; 1 μmol/L) were detected by western blot (^*^P < 0.05 vs. PBS).

To clarify the role of LAPTM5 in pathological cardiac hypertrophy, we detected the expression level of LAPTM5 under various pathological stimuli. According to the western blotting results, the LAPTM5 protein levels in AB-induced hypertrophic mouse hearts were significantly decreased compared with those in sham groups, as assessed by increased levels of cardiac hypertrophic markers, such as atrial natriuretic peptide (ANP) and β-myosin heavy chain (β-MHC) (Figures 1A,B). In addition, we stimulated cardiomyocyte hypertrophy with Ang II and found that ANP and β-MHC levels increased significantly, but the level of LAPTM5 in cardiomyocytes decreased (Figures 1C,D). The above results suggest that the expression of LAPTM5 decreases during myocardial hypertrophy, which may be related to cardiac dysfunction.

### LAPTM5 Suppressed Cardiomyocyte Hypertrophy *in vitro*

**Figure 2:**
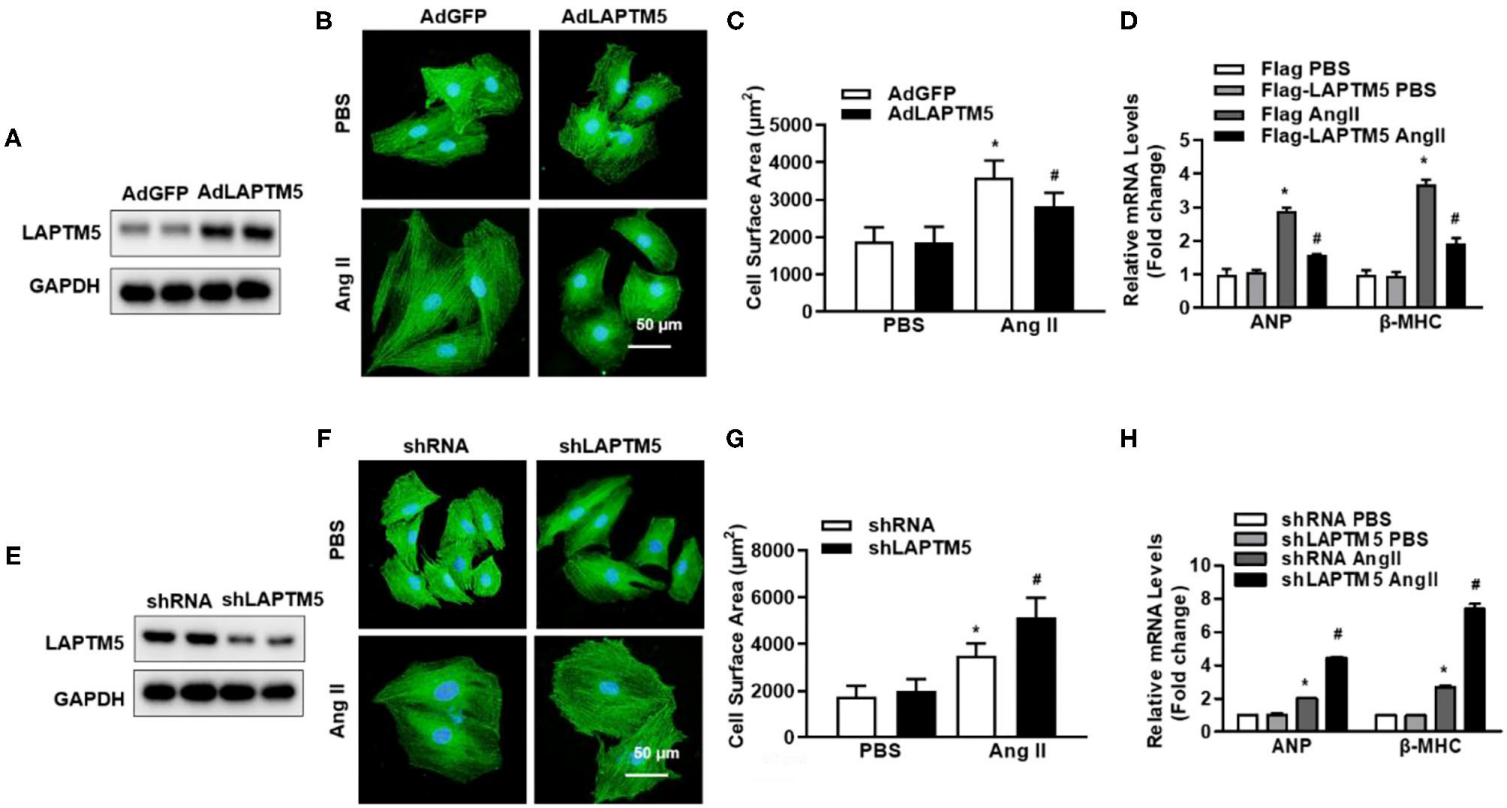
LAPTM5 alleviates cardiomyocyte hypertrophy *in vitro* induced by Ang II. **(A)** Western blot analysis of LAPTM5 expression. **(B)** Representative images of cardiomyocytes infected with the indicated adenoviruses and then treated with PBS or Ang II for 48 h. **(C)** Quantitative results for the cell surface area of NRCMs infected with AdLAPTM5 or AdGFP (control) and treated with PBS or Ang II for 48 h. **(D)** The mRNA levels of ANP and β-MHC in each group (^*^P < 0.05 vs. AdGFP PBS, #*P* < 0.05 vs. AdGFP Ang II). **(E)** The protein levels of LAPTM5 detected by western blot. **(F)** Representative images of cardiomyocytes in the indicated groups. **(G)** Quantitative results for the cell surface area in the indicated groups. **(H)** The mRNA levels of ANP and β-MHC detected by real-time PCR in the indicated groups (^*^P < 0.05 vs. shRNA PBS, ^#^*P* < 0.05 vs. shRNA Ang II).

In the second step, we tried to clarify the regulatory effect of LAPTM5 on pathological cardiac hypertrophy. To confirm this hypothesis, an *in vitro* experiment was performed on NRCMs, and we infected NRCMs with either AdLAPTM5 to overexpress LAPTM5 (Figure 2A) or AdshLAPTM5 to knockdown LAPTM5 (Figure 2E). NRCMs were stimulated with AngII, and cells were transfected with adenovirus to overexpress LAPTM5. With the administration of PBS, there were no significant differences in cardiomyocyte morphology or cardiomyocyte surface area between each group. However, following Ang II stimulation, NRCMs overexpressing LAPTM5 significantly inhibited cardiomyocyte hypertrophy, as assessed by cell surface area (Figures 2B–D). In contrast, there was a significantly greater cardiomyocyte surface area in cells with LAPTM5 knockdown than in AdshRNA-infected controls. In accordance with these results, NRCMs with AdLAPTM5 infection showed reduced ANP and β-MHC transcription levels in response to Ang II, whereas AdshLAPTM5-infected NRCMs showed higher transcription levels of those markers compared with controls under stimulation (Figures 2F–H). Together, these results indicate that LAPTM5 may function as a positive regulator in cardiac hypertrophy.

### Ablation of LAPTM5 Exacerbates Cardiac Remodeling in Mice

**Figure 3:**
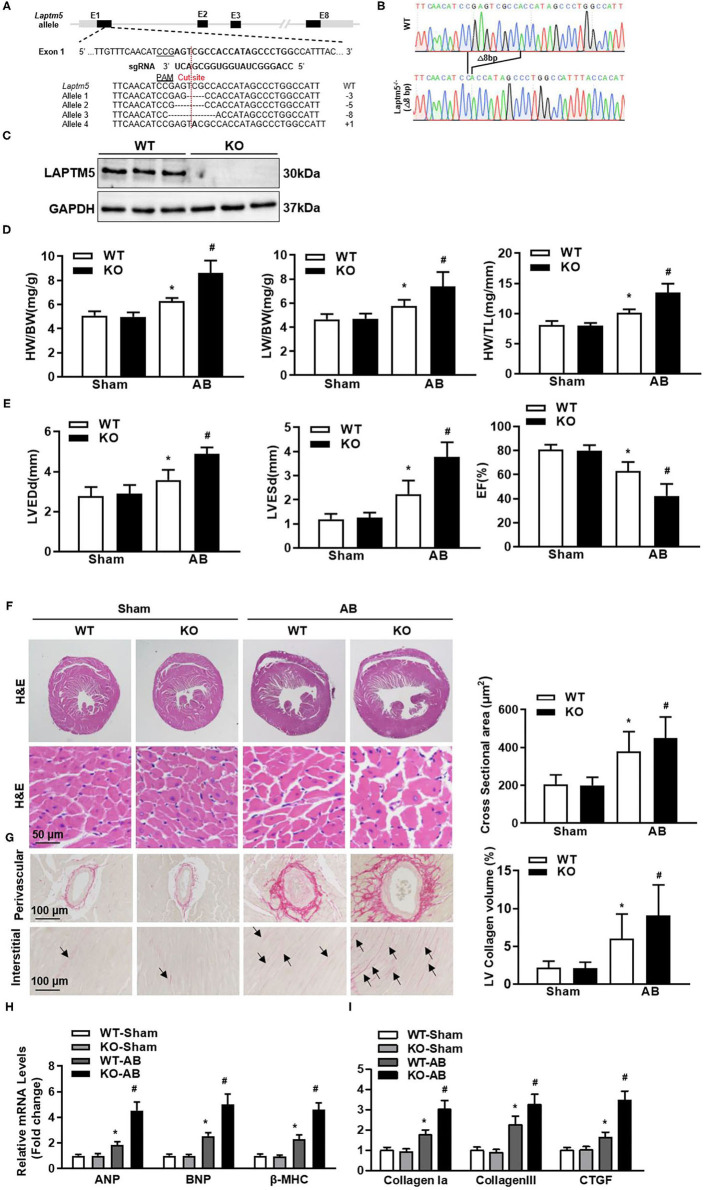
Ablation of LAPTM5 exacerbates pressure overload-induced remodeling. **(A)** Strategy of generating cardiac-specific conditional LAPTM5-KO mice. **(B)** Sanger sequencing results of heart tissue in WT and LAPTM5-KO mice. **(C)** Protein level of LAPTM5 in heart tissue in WT and LAPTM5-KO mice (*n* = 3). **(D)** Heart weight (HW) to body weight (BW) ratio, HW to tibia length (TL) ratio, lung weight (LW) to BW ratio in the indicated groups (*n* = 10). **(E)** Echocardiography results in each group (*n* = 10). LVEDd, left ventricular end diastolic diameter; LVESd, left ventricular end systolic diameter; EF, ejection fraction. **(F)** Representative image of H&E staining and the quantitative results (*n* = 6). **(G)** Representative image of PSR staining and the quantitative result (*n* = 6). **(H,I)** The mRNA levels of hypertrophic and fibrosis markers were detected by real-time PCR (*n* = 6) (^*^P < 0.05 vs. WT-Sham, ^#^*P* < 0.05 vs. WT-AB).

To further confirm the regulatory effect of LAPTM5 on cardiac hypertrophy *in vivo*, we generated LAPTM5 global knockout (KO) mice (Figures 3A–C). Under physiological conditions, LAPTM5 gene deletion mice have healthy behavior and normal fertility, and there is no difference in cardiac morphology and function between LAPTM5 gene deletion mice and WT mice. After 4 weeks of AB, LAPTM5 gene-deleted mice showed worsening pathological myocardial hypertrophy, as assessed by higher heart weight (HW)/body weight (BW), lung weight (LW)/BW and HW/tibial length (TL) (Figure 3D) than WT controls. In addition, LAPTM5 gene-deleted mice showed more severe cardiac dilation and dysfunction, as evidenced by echocardiographic parameters (Figure 3E). The staining results showed that the cross-sectional area of cardiomyocytes in LAPTM5 gene-deleted mice was larger than that in WT mice (Figure 3F). In addition, the transcription of cardiac hypertrophy markers in LAPTM5-ko mice was significantly increased (Figure 3H).

Myocardial fibrosis is also an important sign of pathological cardiac hypertrophy (17). Therefore, we examined the effect of LAPTM5 gene deletion on myocardial fibrosis. The staining results showed obvious perivascular and interstitial fibrosis in WT mice 4 weeks after AB operation. However, this fibrosis was significantly aggravated in the hearts of gene-deleted mice (Figure 3G). LAPTM5 gene deletion also increased the transcription of fibrosis markers (collagen I, collagen III and connective tissue growth factor) (Figure 3I). These results suggest that LAPTM5 gene deletion can aggravate the pathological myocardial hypertrophy induced by pressure overload.

### LAPTM5 Overexpression Relieved Cardiac Remodeling in Mice

**Figure 4:**
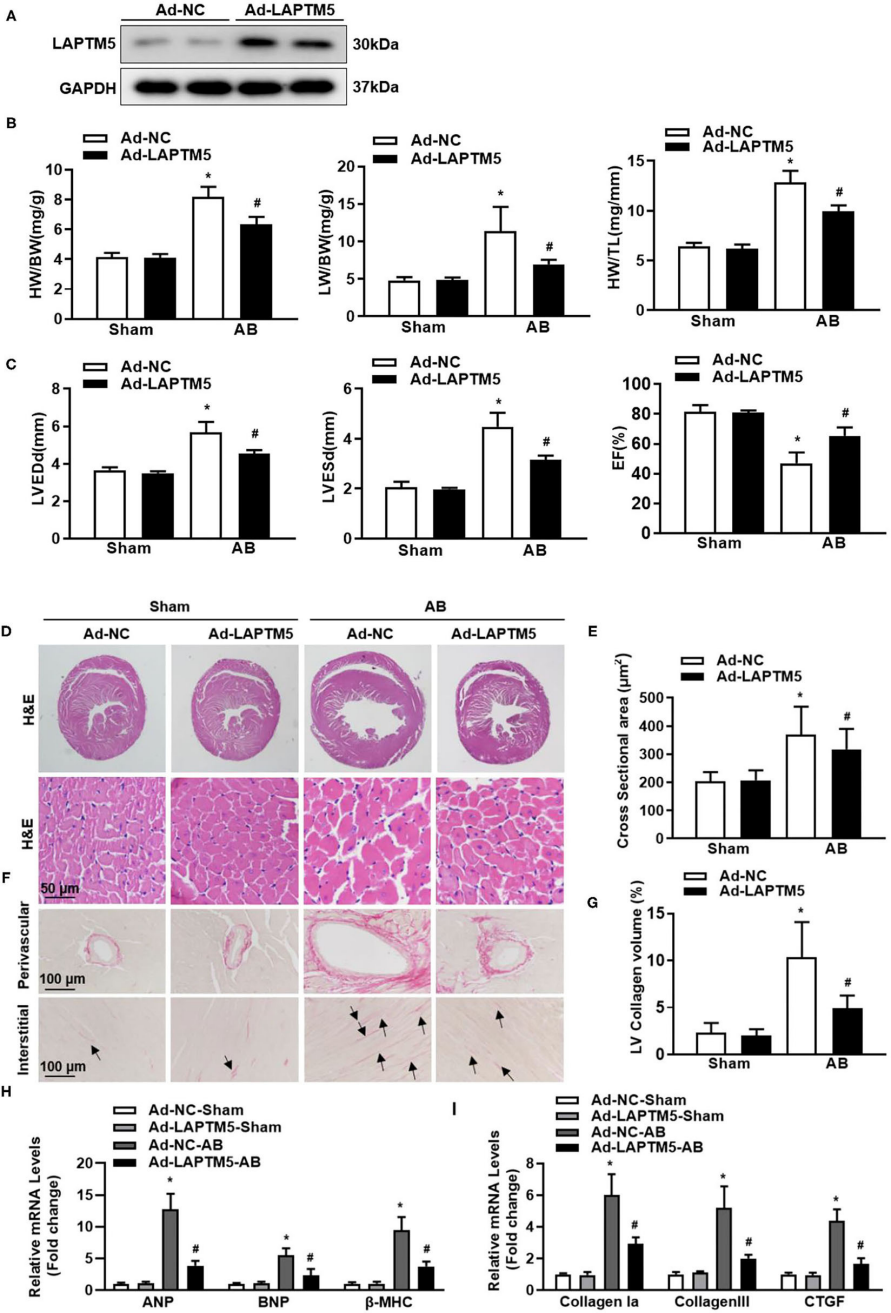
Overexpression of LAPTM5 attenuates AB-induced cardiac hypertrophy. **(A)** Protein level of LAPTM5 in heart tissue in mice injected with Ad-NC or Ad-LAPTM5 (*n* = 3). **(B)** Heart weight (HW) to body weight (BW) ratio, HW to tibia length (TL) ratio, lung weight (LW) to BW ratio in the indicated groups (*n* = 11–15). **(C)** Echocardiography results in the indicated groups (*n* = 8). **(D,E)** Representative images of H&E staining and the quantitative results (*n* = 6). **(F,G)** Representative image of PSR staining and the quantitative result (*n* = 6). **(H,I)** The mRNA levels of hypertrophic and fibrosis markers (*n* = 6) (^*^P < 0.05 vs. Ad-NC-Sham, ^#^*P* < 0.05 vs. Ad-NC-AB).

Since AAV9 has strong guidance to cardiomyocytes (18), we used the AAV9 overexpression system to overexpress LAPTM5 in mouse hearts. Mice injected with AAV9-LAPTM5 showed elevated expression of LAPTM5 compared with the control mice (Figure 4A). Moreover, LAPTM5 overexpression suppressed the cardiac hypertrophic response, as we observed reduced HW/BW, HW/TL, and LW/BW ratios in the AAV9-LAPTM5 group 4 weeks after AB (Figure 4B). Mice injected with AAV9-LAPTM5 also showed ameliorated cardiac dilation and dysfunction (Figure 4C), a lower cardiomyocyte cross-sectional area (Figures 4D,E), and reduced transcription of hypertrophic markers compared with the control mice (Figure 4H).

Consistently, cardiac fibrosis was also attenuated in mice injected with AAV9-LAPTM5 (Figures 4F,G), as assessed by PSR staining and analysis of collagen volume and fibrosis marker expression (Figure 4I). Together, these data suggest that LAPTM5 protects against cardiac hypertrophy and heart failure.

### LAPTM5 Suppressed MEK-ERK1/2 Signaling Pathway

**Figure 5:**
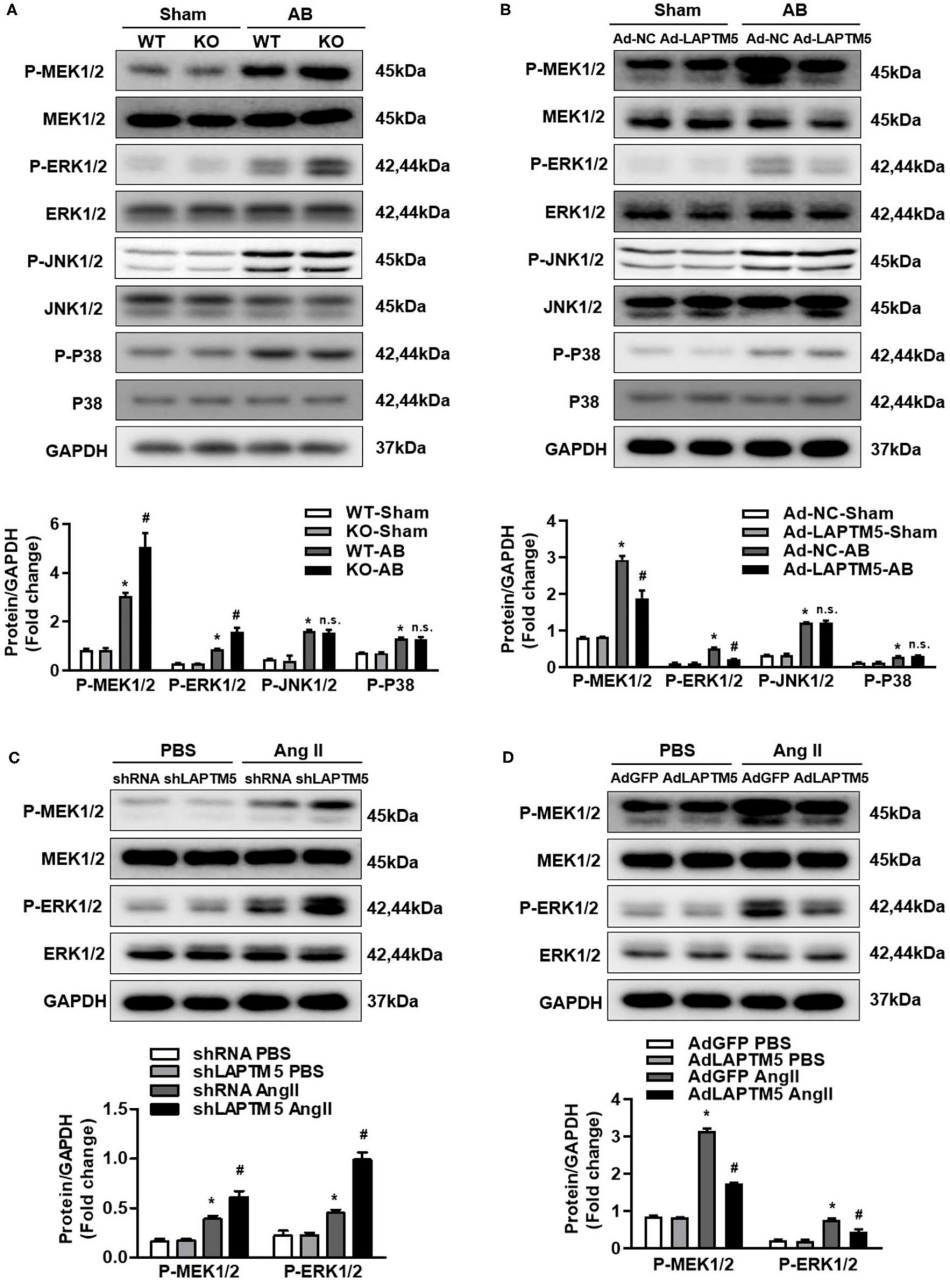
LAPTM5 inhibits the pressure overload-mediated MEK-ERK1/2 signaling pathway. **(A)** Protein levels of phosphorylated (p-) and total (T-) MEK1/2, ERK1/2, JNK1/2, and P38 in heart tissue in WT and LAPTM5-KO mice (*n* = 3) (^*^P < 0.05 vs. WT-Sham, ^#^*P* < 0.05 vs. WT-AB). **(B)** Protein levels of phosphorylated and total MEK1/2, ERK1/2, JNK1/2, and P38 in heart tissue in mice injected with Ad-NC or Ad-LAPTM5 (*n* = 3) (^*^P < 0.05 vs. Ad-NC-Sham, ^#^*P* < 0.05 vs. Ad-NC-AB). **(C)** Protein levels of phosphorylated and total MEK1/2 and ERK1/2 in NRCMs transfected with shLAPTM5 (*n* = 3) (^*^P < 0.05 vs. shRNA PBS, ^#^*P* < 0.05 vs. shRNA Ang II). **(D)** Protein levels of phosphorylated and total MEK1/2 and ERK1/2 in NRCMs transfected with Flag-LAPTM5 (*n* = 3) (^*^P < 0.05 vs. AdGFP PBS, ^#^*P* < 0.05 vs. AdGFP Ang II).

Because the MAPK pathway plays a key role in pathological cardiac hypertrophy, we examined whether LAPTM5 affects these signaling molecules (19, 20). After 4 weeks of AB, the phosphorylated MEK1/2 and ERK1/2 in the hearts of WT mice increased significantly, but the phosphorylation level of the above proteins in the hearts of LAPTM5 gene-deleted mice was higher than that in the WT group (Figure 5A). The phosphorylation level of the above proteins in the hearts of LAPTM5-overexpressing mice was lower than that in the hearts of control mice (Figure 5B). However, deletion or overexpression of LAPTM5 in mouse hearts did not affect the levels of phosphorylated JNK1/2 and p38 (Figures 5A,B). To further verify these results, we detected changes in MAPK signaling molecules in NRCMs. Our results showed that silencing LAPTM5 in NRCMs could increase the phosphorylation levels of MEK1/2 and ERK1/2 (Figure 5C). However, overexpression of LAPTM5 in cardiomyocytes reduced the phosphorylation levels of MEK1/2 and ERK1/2 (Figure 5D). These results suggest that LAPTM5 inhibits the activation of MEK1/2 and ERK1/2 signaling and plays a cardioprotective role.

### LAPTM5 Regulates Rac1 Through Direct Physical Interaction

**Figure 6:**
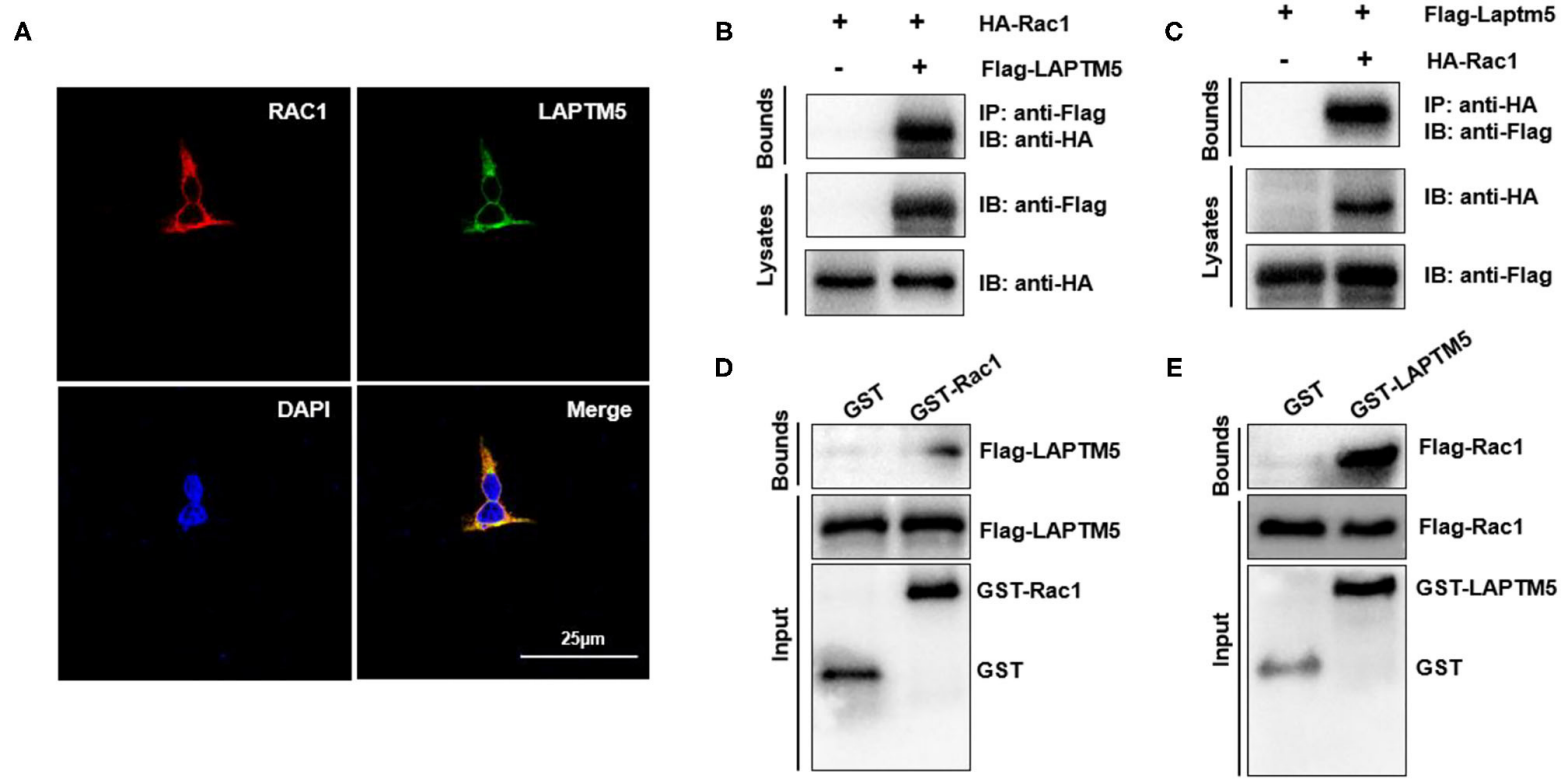
LAPTM5 regulates Rac1 through direct physical interaction. **(A)** The colocalization of Rac1 and LAPTM5 in 293T cells. The staining of LAPTM5 is green, Rac1 is red and DAPI is blue in representative confocal images. **(B,C)** The Rac1 and LAPTM5 interaction was detected by immunoprecipitation (IP) assays. **(D,E)** The direct interaction between Rac1 and LAPTM5 was verified by GST pull-down assays.

The above results indicated that LAPTM5 might regulate pressure overload-induced cardiac hypertrophy by inhibiting MEK-ERK signaling. However, the molecular mechanisms of how LAPTM5 regulates the MEK-ERK signaling pathway remain unknown. Rac1 is one of the most upstream regulators of MAPK signaling and has been reported to localize to endosomes, which are also located in the region of LAPTM5 (21). To further validate the correlation between LAPTM5 and Rac1, we first examined the subcellular localization of LAPTM5 and Rac1 by immunofluorescent staining. 293T cells were transfected with pEGFP-LAPTM5 and pmCherry-Rac1. Both LAPTM5 and Rac1 were located in the cytoplasm, and their locations overlapped with each other (Figure 6A). We also detected the interaction between LAPTM5 and Rac1. 293T cells were transfected with Flag-LAPTM5 and HA-Rac1. Coimmunoprecipitation results showed an interaction of Flag-LAPTM5 with HA-Rac1 (Figures 6B,C). We also GST pulldown to confirm this interaction. The results revealed that Flag-LAPTM5 was pulled down with GST-tagged Rac1, and HA-Rac1 was pulled down with GST-tagged LAPTM5 but not GST per se (Figures 6D,E). Overall, these data demonstrated that LAPTM5 could directly interact with Rac1.

### LAPTM5 Suppresses the MEK-ERK1/2 Signaling Pathway *via* Rac1

**Figure 7:**
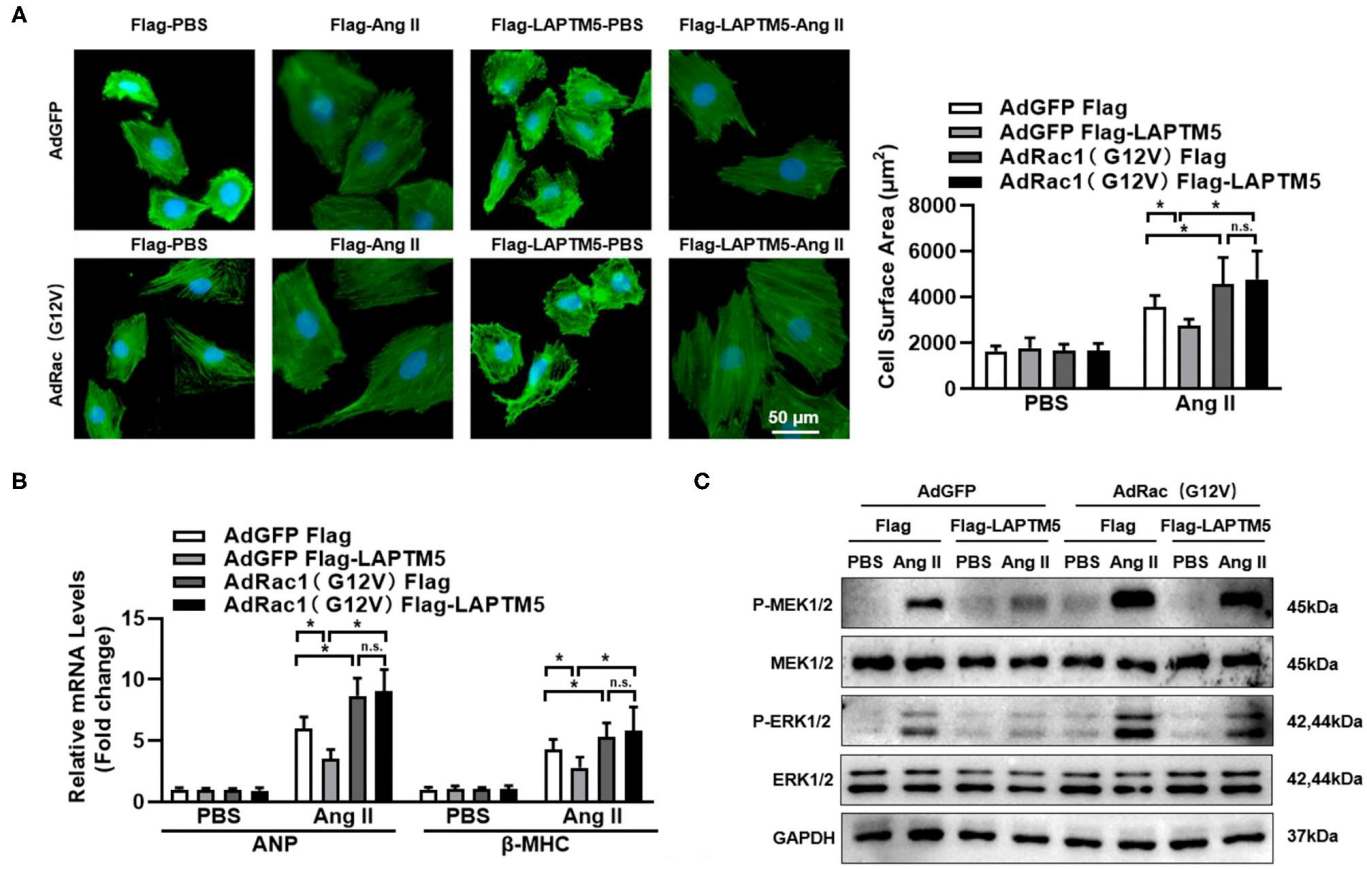
LAPTM5 suppresses the MEK-ERK signaling pathway *via* Rac1. **(A–C)** NRCMs were overexpressed with a constitutively active mutant of Rac1 (G12V) and Flag-LAPTM5 or Flag (control) and treated with Ang II for 12 h. **(A)** α-actinin staining and quantitative results for the cross-sectional area (*n* = 6). **(B)** mRNA level of ANP and BNP (^*^P < 0.05 vs. Flag-PBS, #*P* < 0.05 vs. Flag-Ang II). **(C)** Protein levels of phosphorylated (p-) and total (T-) MEK1/2 and ERK1/2 (*n* = 6).

We further explored whether activation of the MEK-ERK signaling cascade by Rac1 could affect the regulatory effect of LAPTM5 in cardiomyocyte hypertrophy. We overexpressed a constitutively active mutant of Rac1 (G12V) in NRCM (22), and the western blot results revealed that Ang II-triggered activation of MEK1/2 and ERK1/2 was exaggerated in Rac1 (G12V)-expressing cardiomyocytes. In response to Ang II induction, the protective effect of LAPTM5 on cardiomyocyte hypertrophy was markedly blocked by Rac1 (G12V) overexpression (Figure 7A). Furthermore, the mRNA levels of hypertrophic markers were altered in a similar pattern (Figure 7B). In accordance with the above results, Rac1 (G12V) expression depleted the attenuating effects of LAPTM5 on the phosphorylation levels of MEK1/2 and ERK1/2 (Figure 7C). Taken together, these results demonstrate that LAPTM5 ameliorates the development of pathological cardiac hypertrophy by interacting with Rac1 and then blocking the activation of the Rac1-dependent signaling cascade.

## Discussion

LAPTM5, a lysosomal protein that is highly expressed in cells of lymphoid and myeloid origin, functions in immune cells and the inflammatory response (11). In T cells, LAPTM5 promotes lysosomal translocation of intracellular CD3ζ, which regulates T cell function (23). LAPTM5 also participates in HeLa cell apoptosis *via* cleavage of Mcl-1 and Bid, which activate mitochondria-dependent caspase apoptosis (9). Herein, we demonstrated that LAPTM5 was also expressed in cardiomyocytes and downregulated under hypertrophy insults. We used LAPTM5 knockout mice and found that LAPTM5 deficiency deteriorated the cardiac hypertrophic response and cardiac dysfunction induced by pressure overload. In addition, we also revealed that LAPTM5 regulated MEK-ERK1/2 activation by inhibiting the endosome protein Rac1. Additionally, we supported the protective role of LAPTM5 in cardiac hypertrophy using a LAPTM5-overexpressing AAV9 delivery system *in vivo*.

Pathological cardiac hypertrophy is usually caused by pressure stimulation. It is a typical pathological stage of cardiovascular disease, such as cardiomyopathy, myocardial infarction and diabetes (24). Pathological cardiac hypertrophy is a risk factor for cardiovascular disease-caused death. Pathological cardiac hypertrophy is characterized by a large LV mass, LV fibrosis and decreased systolic and diastolic function (25). LAPTM5 was reported to regulate T and B cell receptor signaling and the human cancer cell cycle and death (9, 23). The effect of LAPTM5 remains unknown. In our study, we found that in both LV tissues of mice and isolated NRCMs, LAPTM5 was downregulated at the decompensation stage in response to hypertrophic stimulus. Activation of LAPTM5 may be beneficial for cardiomyocytes to resist pathological stimuli. When we knocked out LAPTM5 in mice, the pathological cardiac hypertrophy phenotype became even worse with larger LV mass, fibrosis and cardiac dysfunction. In addition, cardiac LAPTM5 overexpression by AAV9 injection ameliorated pressure overload-induced cardiac hypertrophy. These results suggest that a higher level of LAPTM5 in cardiomyocytes might retard the transformation of hypertrophy to heart failure.

**Figure 8:**
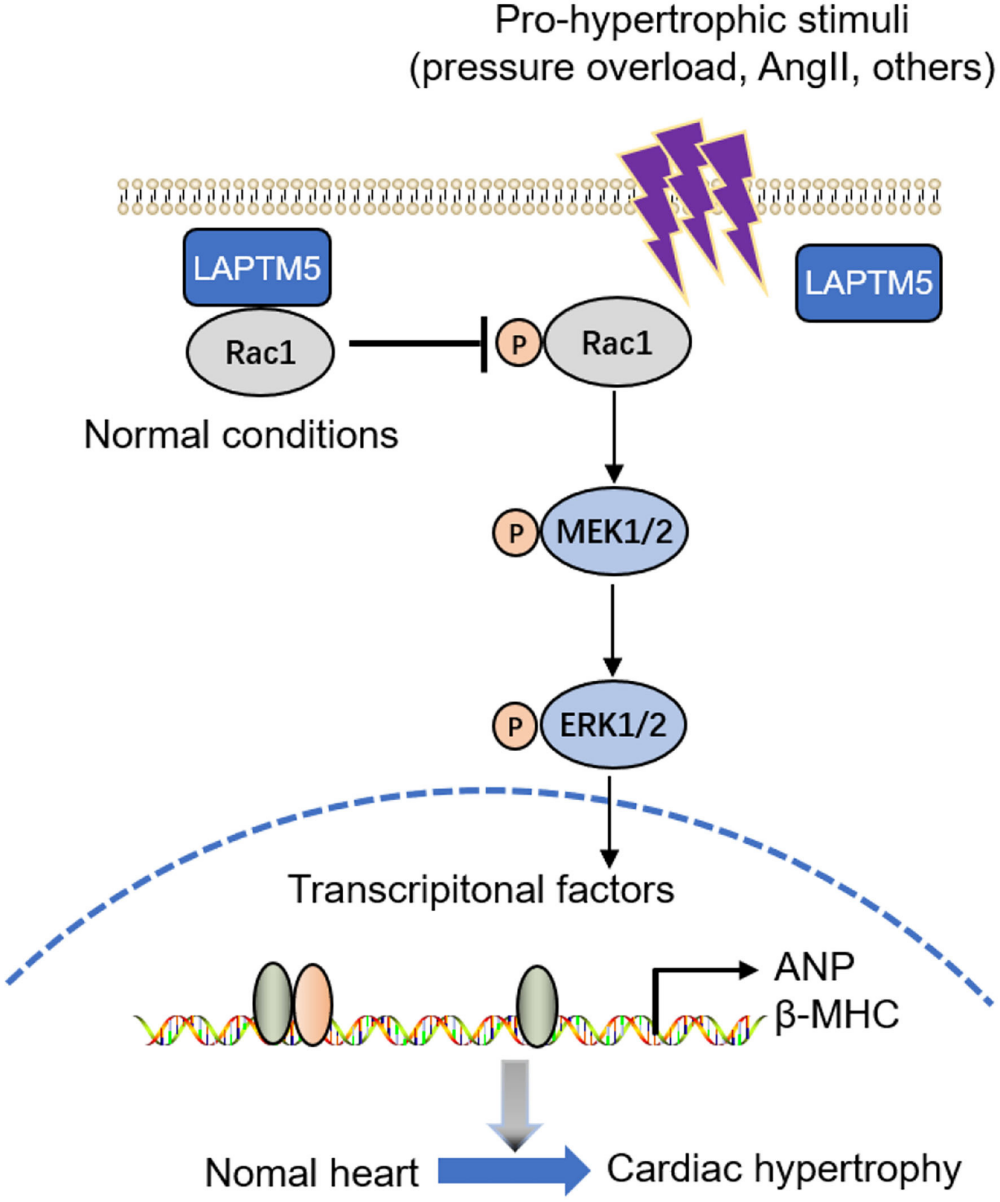
Graphical representation of the role and signaling of LAPTM5 in cardiac hypertrophy. LAPTM5 binds to Rac1, inhibiting cardiac hypertrophy by suppressing the MEK-ERK1/2 pathway.

Extracellular signal-regulated kinases (ERKs), c-Jun N-terminal kinases (JNKs) and p38 all belong to MAPK signaling (26). MAPK plays an important role in mediating myocardial hypertrophy induced by overload or pathological stimulation. Cardiomyocyte-specific overexpression of MEK-1/2 promotes the phosphorylation and activation of ERK1/2, inducing cardiomyocyte hypertrophic growth and cardiac central hypertrophy (27). The ERK1/2 pathway is involved in the regulation of cardiomyocyte growth, while JNK and p38 signaling are involved in myocardial stress responses, such as oxidative stress, osmotic shock, infection and cytokines (28). Cardiac-specific ERK1/2 knockout mice and ERK1/2 heterozygous mice can resist pressure overload-induced cardiac hypertrophy, apoptosis and heart failure (29). Our results further supported that LAPTM5 overexpression may attenuate the development of pathological cardiac hypertrophy by blocking the activation of MEK 1/2 and ERK1/2. To gain insights into the mechanisms by which LAPTM5 regulated the MEK-ERK1/2 signaling pathway, we performed mass spectrometry analysis and identified Rac1, a GTP-binding protein. Numerous studies have confirmed that Rac1 is involved in pressure overload-induced heart failure and the MEK-ERK1/2 signaling pathway (30). In our study, Rac1 was found to be the direct target of LAPTM5 in cardiomyocytes, as LAPTM5 overlaps with the Rac1 protein and interacts with each other. Then, we explored the effects of Rac1 (G12V) on the inactivation of MEK1/2 and ERK1/2 by LAPTM5. Our results showed that Rac1 (G12V) can eliminate the inactivation of MEK1/2 and ERK1/2 by LAPTM5, indicating that the inhibitory role of STEAP3 in the MEK-ERK signaling pathway seemed to be largely dependent on Rac1 (Figure 8). However, additional studies are needed to decipher the exact mechanisms involved in modulating Rac1 activity by LAPTM5.

A previous study found that LAPTM5 functions mainly in immune cells and cancer cells (11). In this study, we provide evidence that LAPTM5 also plays an important role in cardiac hypertrophy *via* the Rac1-MEK-ERK1/2 pathway. Although our study suggested that LAPTM5 binds to Rac1, the fact that LAPTM5 induces Rac1 lysosomal degradation or protein modification is still unclear. The mechanism by which LAPTM5 is downregulated during cardiac hypertrophic progression in cardiomyocytes is also unknown. Thus, further studies are needed to explore this unsolved puzzle. In conclusion, our study enriches the mechanism of pathological myocardial hypertrophy. It is of far-reaching significance to explore molecular therapies targeting LAPTM5 for the treatment of heart failure.

## Data Availability Statement

The original contributions presented in the study are included in the article/Supplementary Material, further inquiries can be directed to the corresponding author/s.

## Ethics Statement

The animal study was reviewed and approved by the Animal Care and Use Committee of the First Affiliated Hospital of Zhengzhou University.

## Author Contributions

LG, SG, and RL contributed to the conception and design of the experiments. LG, RY, and XZ carried out the experiments. YZ and LX analyzed the experimental results. LG and XW wrote and revised the manuscript. All authors contributed to the article and approved the submitted version.

## Funding

This study was supported by the National Natural Science Foundation of China (Grant Nos. 81970201, 82070284) and the Outstanding Youth Science Fund of Henan Province (Grant No. 212300410076).

## Conflict of Interest

The authors declare that the research was conducted in the absence of any commercial or financial relationships that could be construed as a potential conflict of interest.

## Publisher's Note

All claims expressed in this article are solely those of the authors and do not necessarily represent those of their affiliated organizations, or those of the publisher, the editors and the reviewers. Any product that may be evaluated in this article, or claim that may be made by its manufacturer, is not guaranteed or endorsed by the publisher.
