# Supplementary material for: Lysosomal-Associated Protein Transmembrane 5 Functions as a Novel Negative Regulator of Pathological Cardiac Hypertrophy
Source: Front Cardiovasc Med. 2021 Oct 6;8:740526. doi: 10.3389/fcvm.2021.740526 (PMC8526538; doi:10.3389/fcvm.2021.740526)

Figure 1A

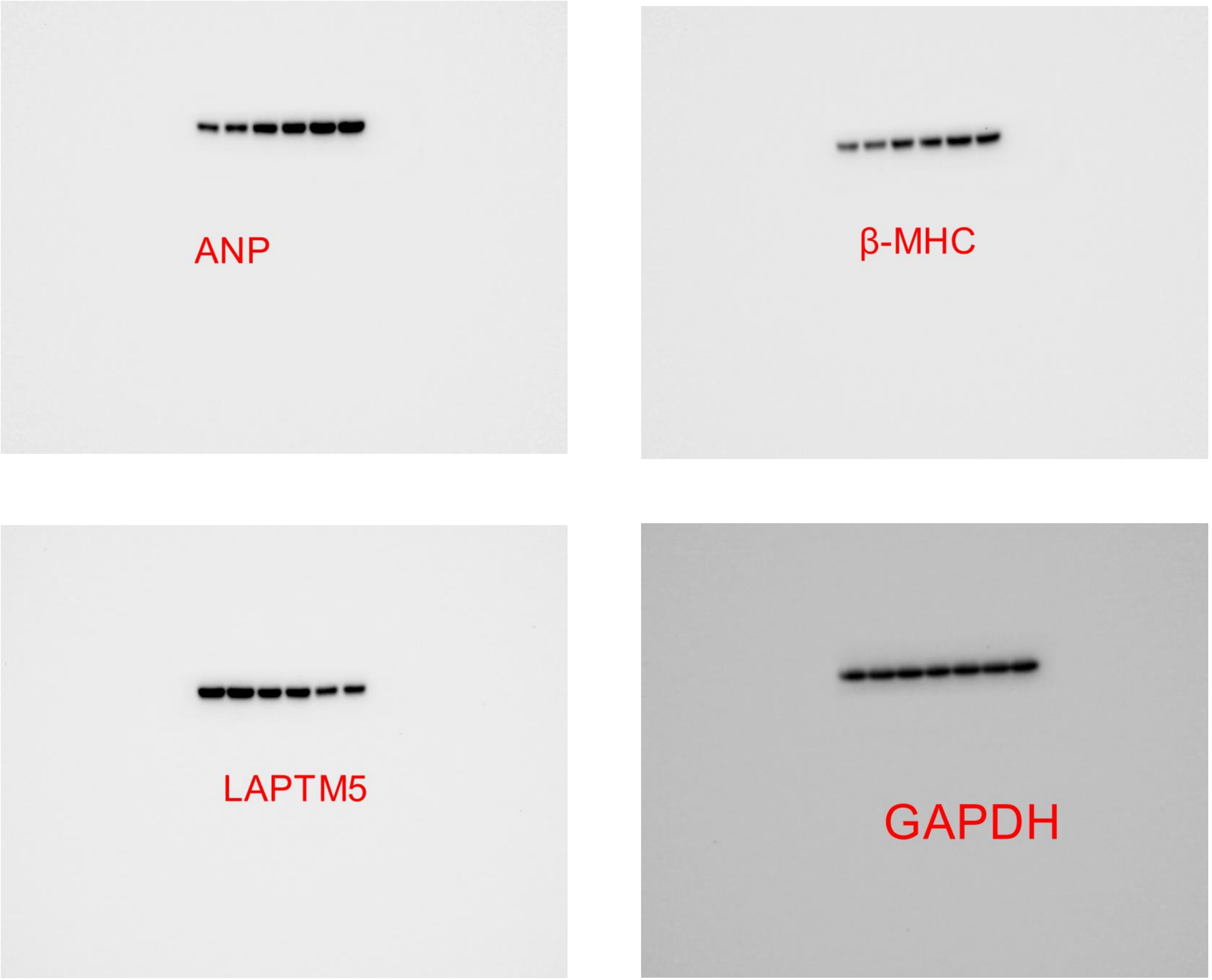

Figure 1B

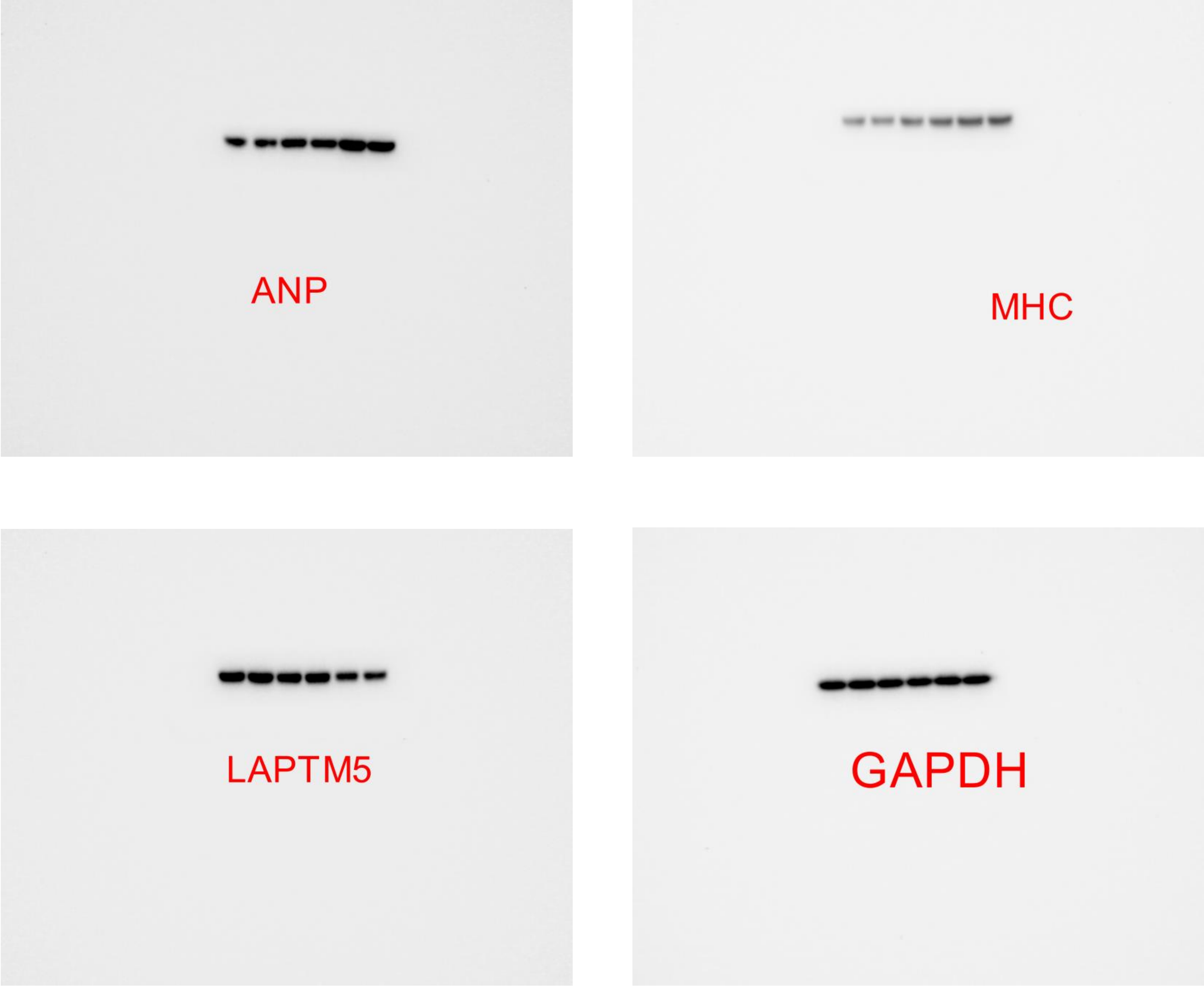

Figure 2A

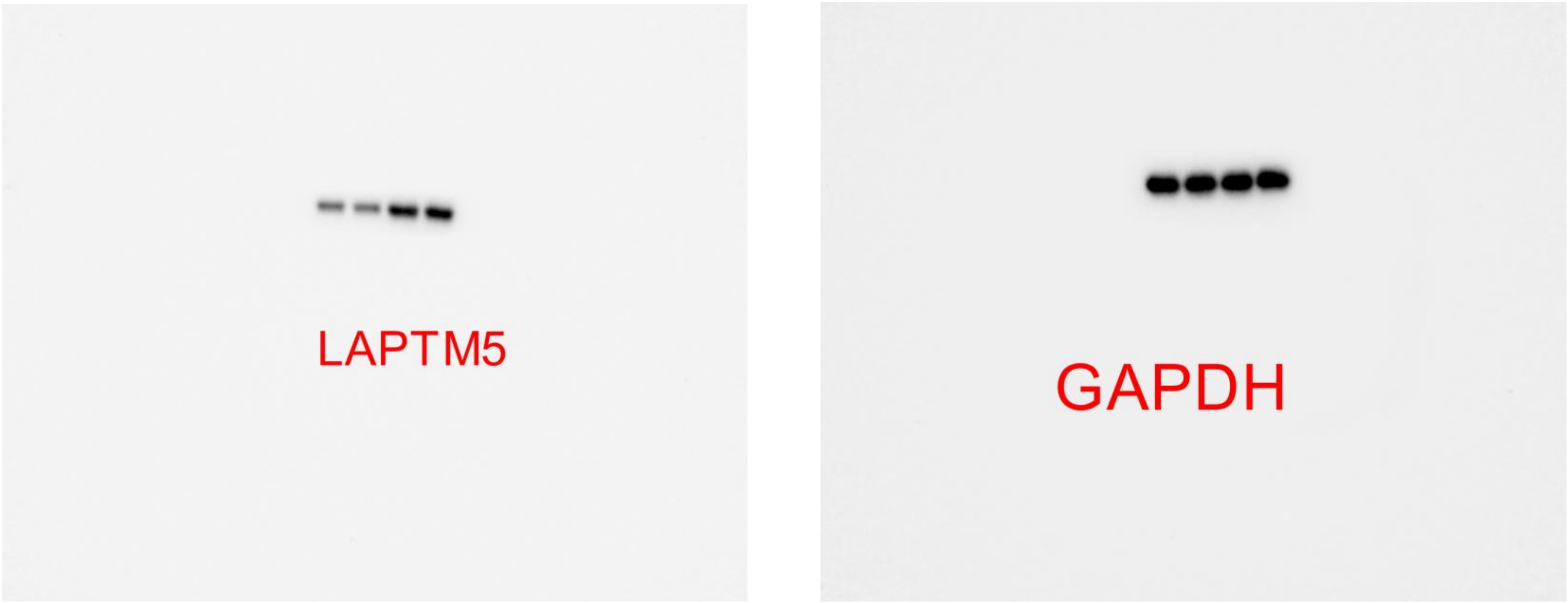

Figure 2E

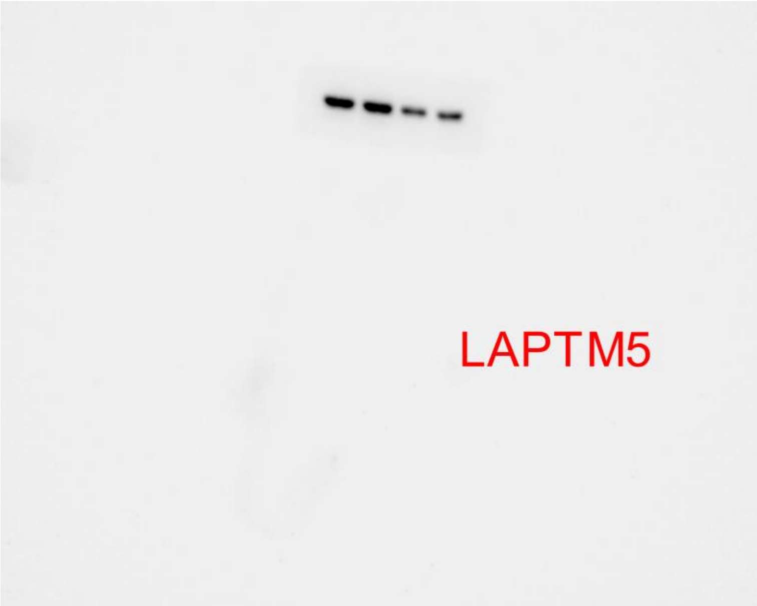

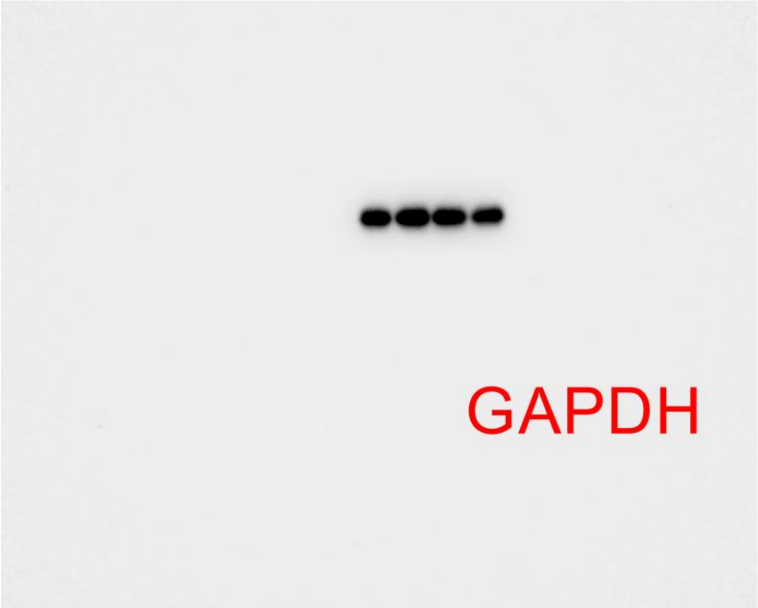

Figure 3C

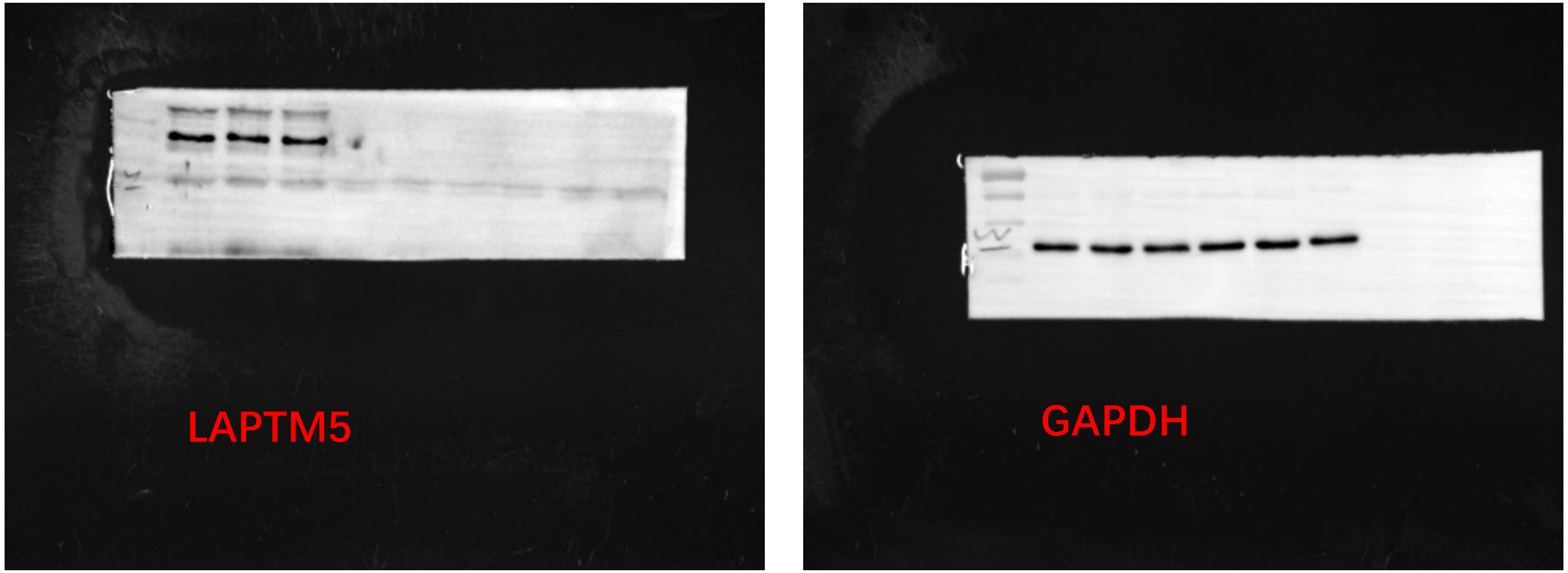

Figure 4A

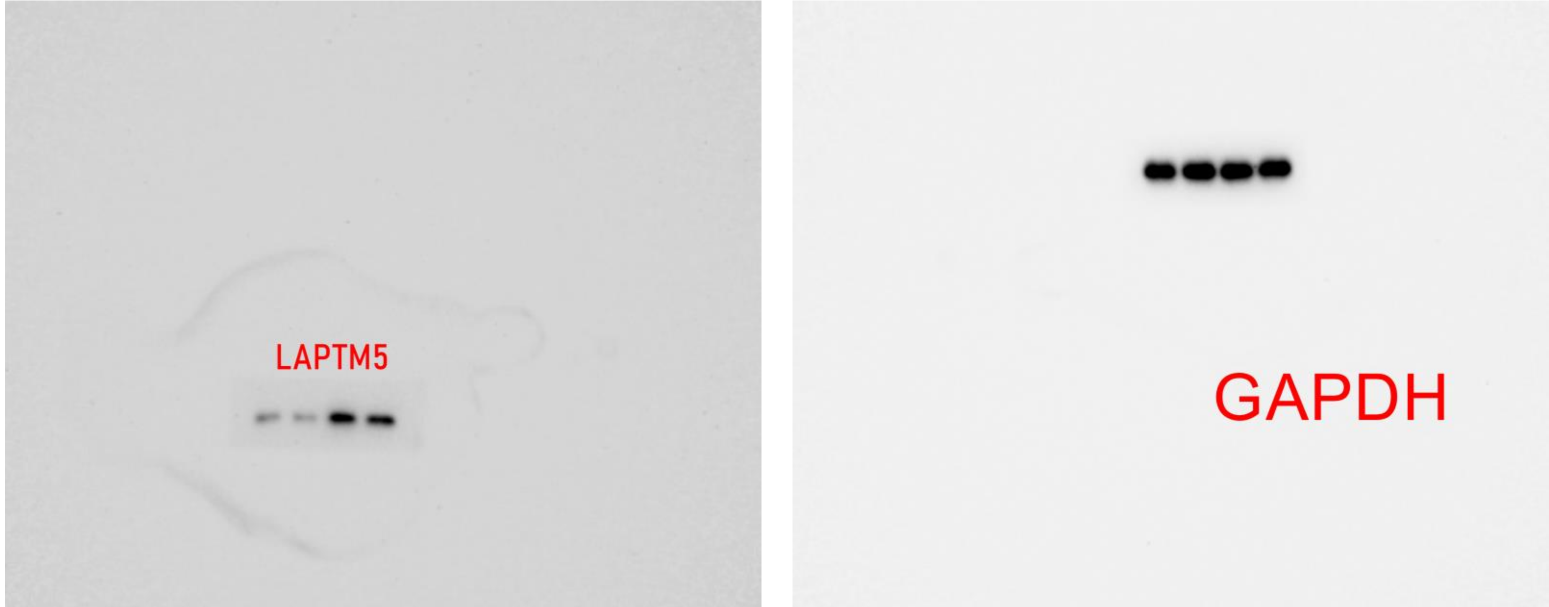

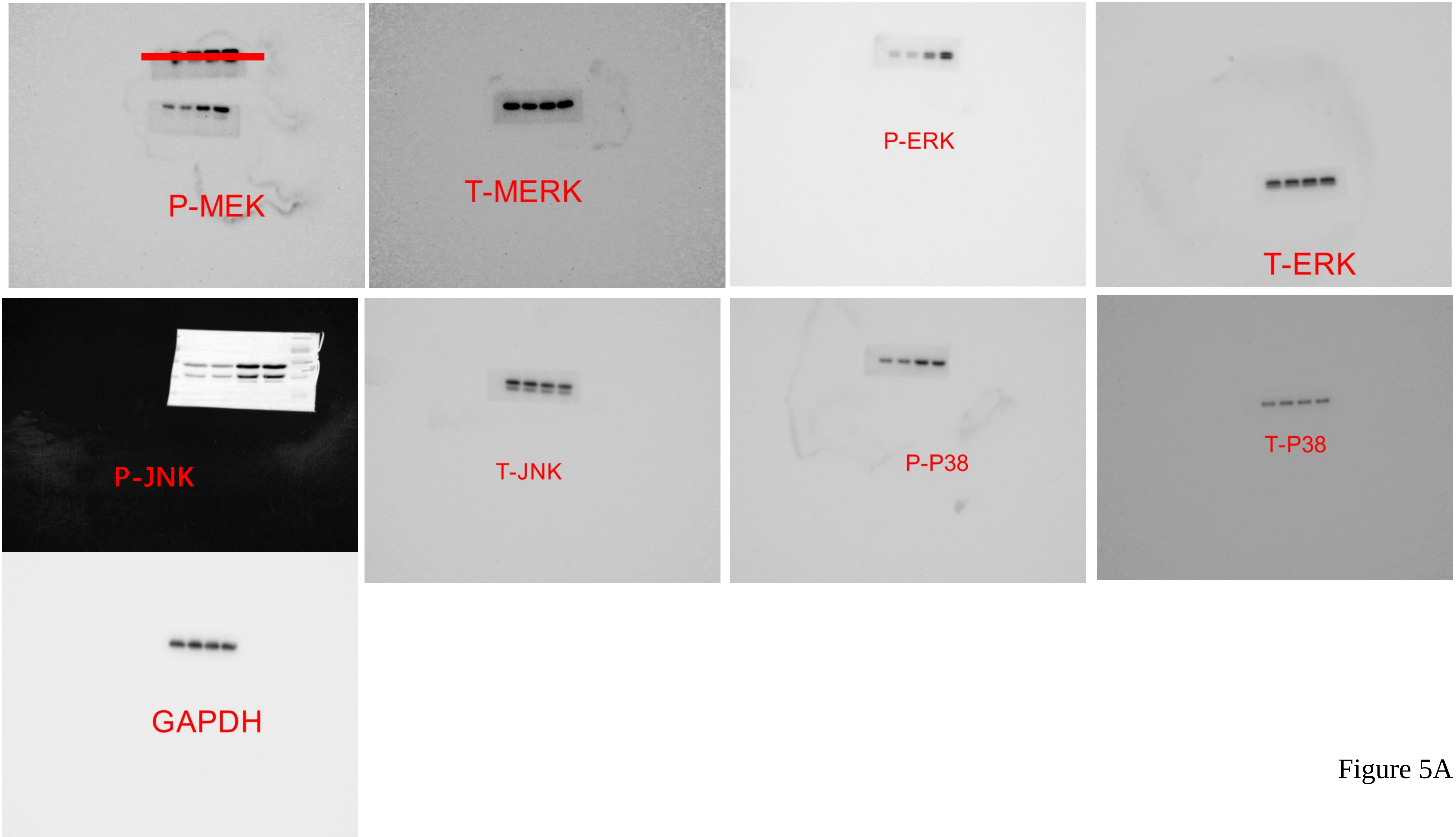

Figure 5A

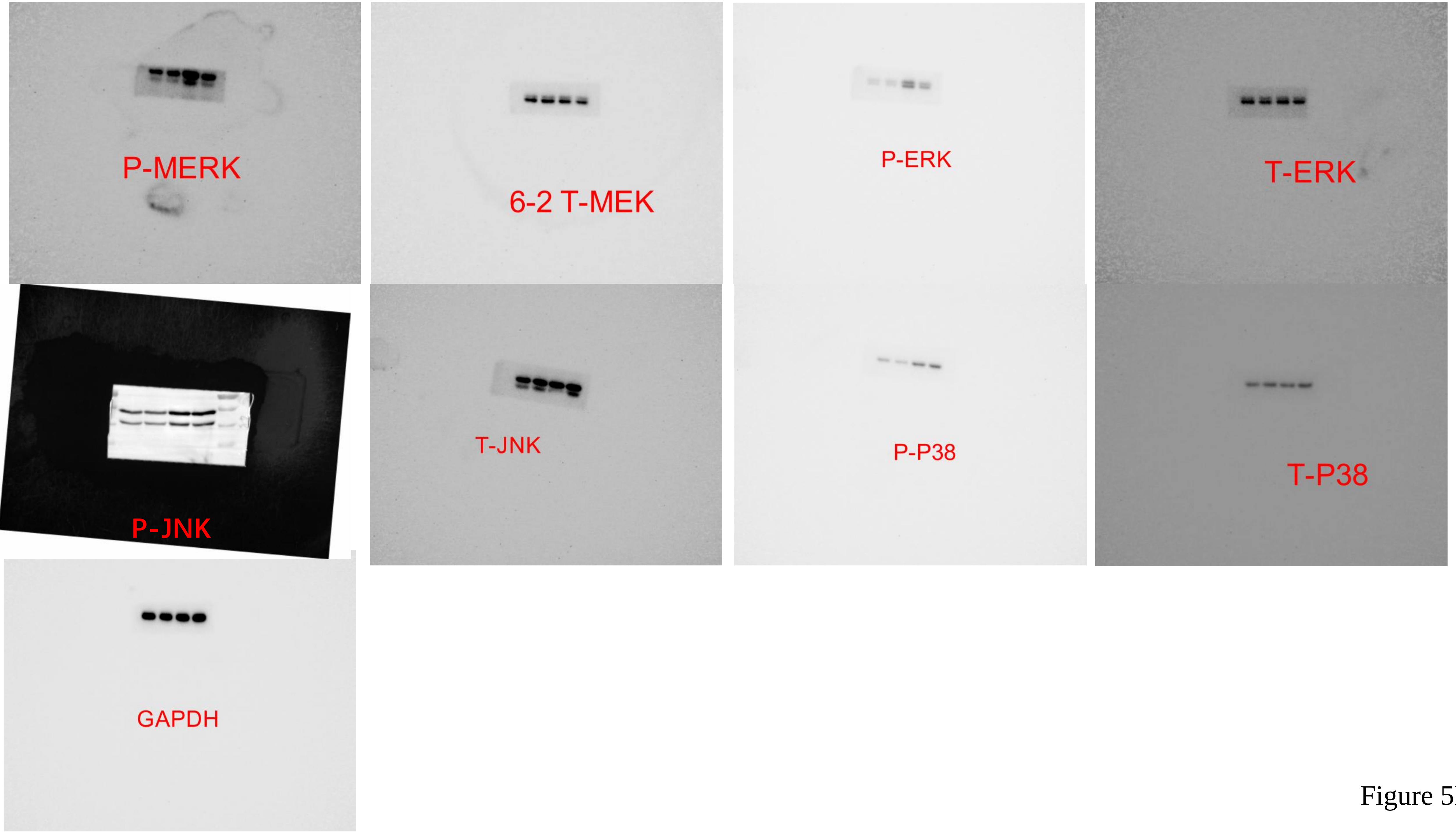

Figure 5B

Figure 5C

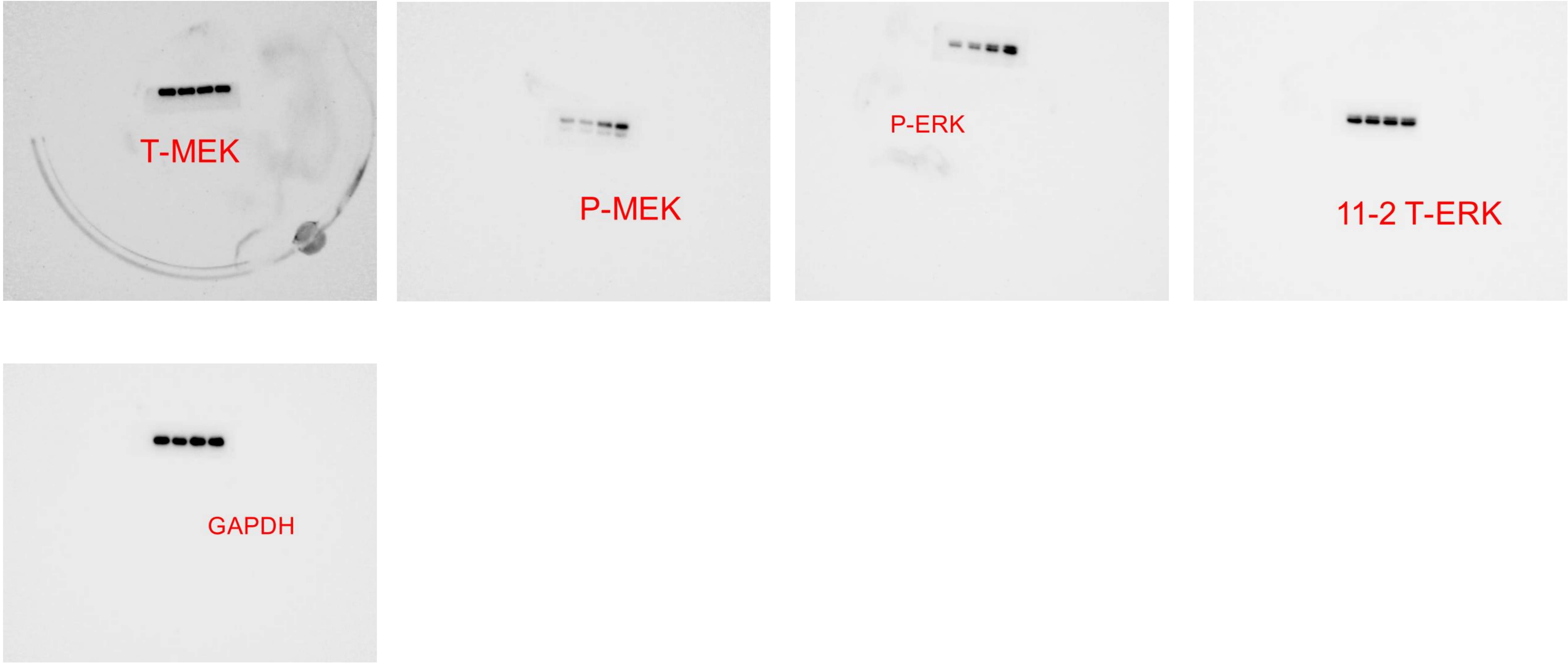

Figure 5D

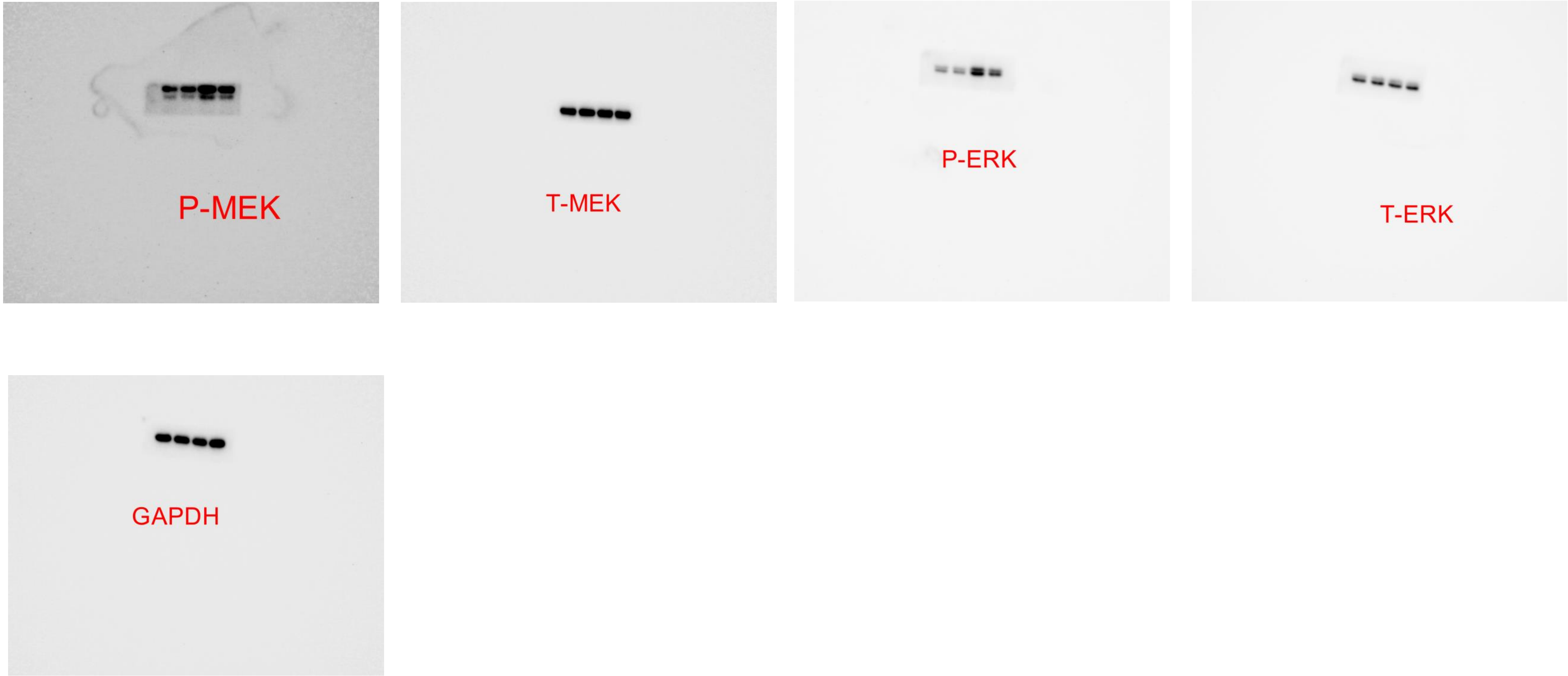

Figure 6B

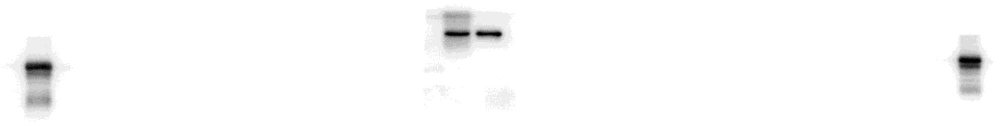

IN-FLAG IN-HA IP-HA

Figure 6C

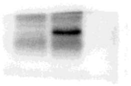

IN-HA

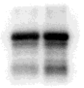

IN-FALG

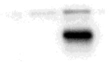

IP-FLAG

Figure 6D

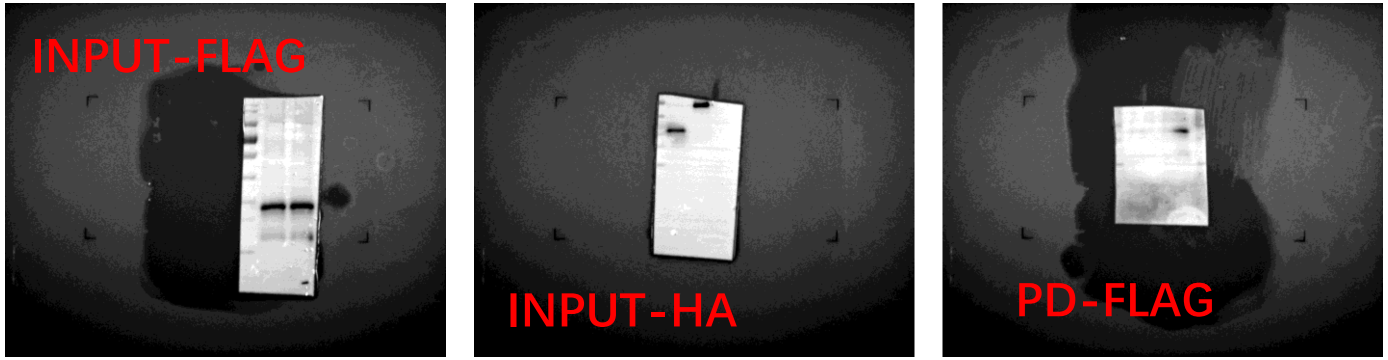

Figure 6E

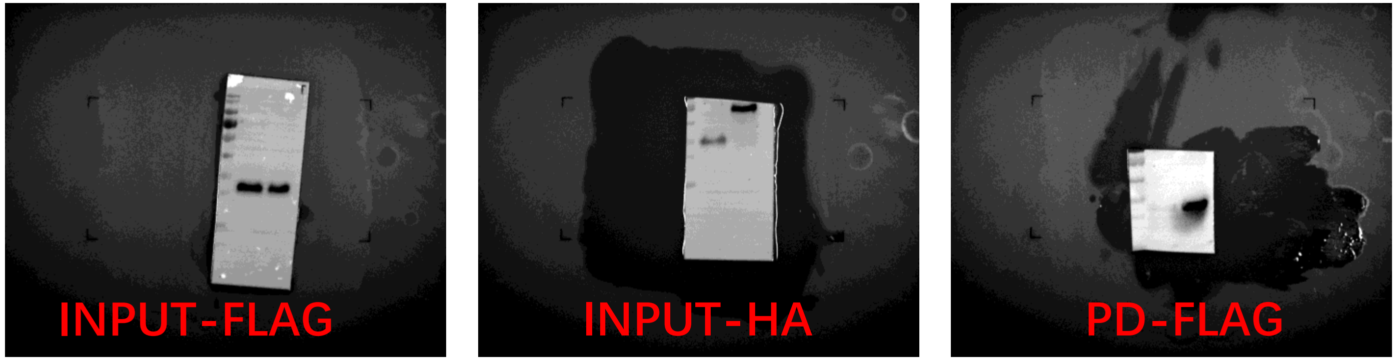

Figure 7C

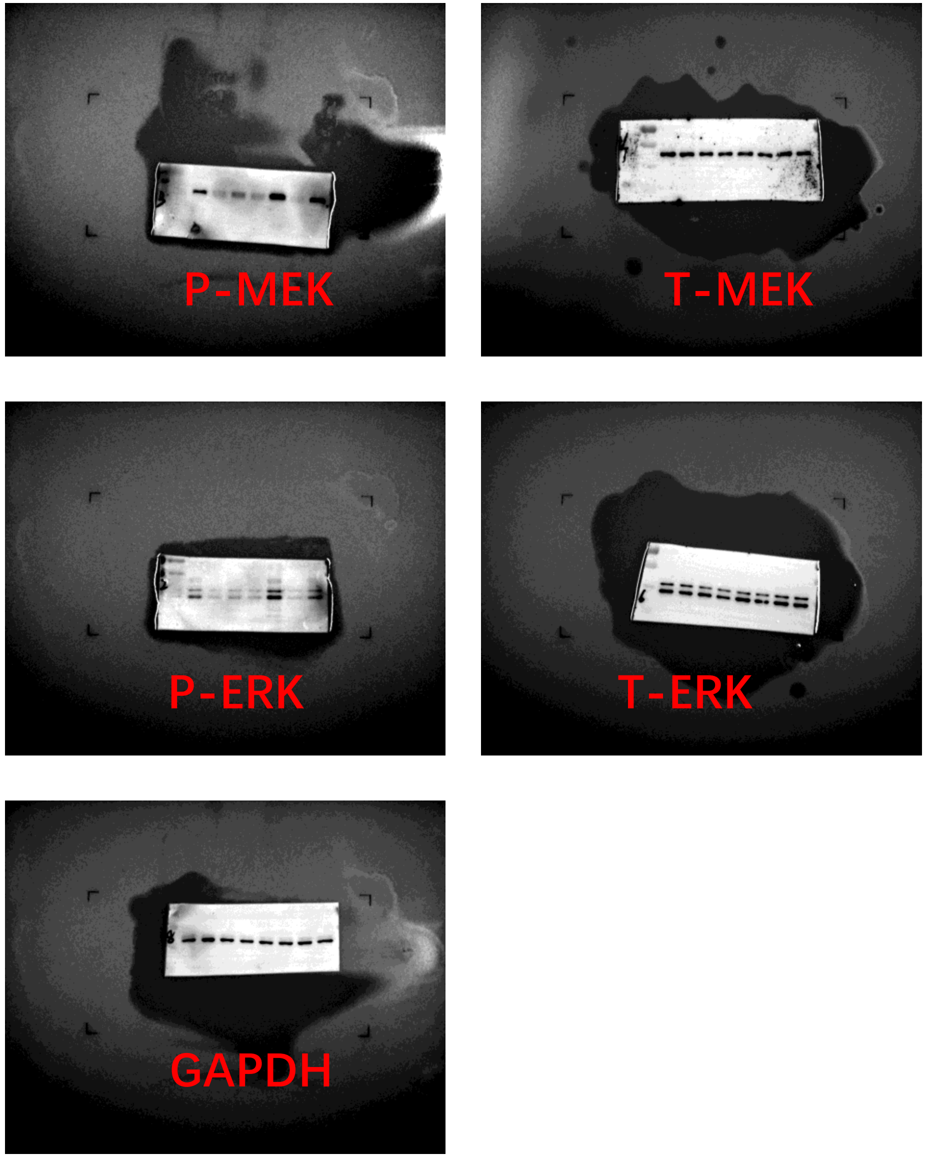

Supplement: Supplementary file 1 [file Data_Sheet_1.PDF]
